# Transcriptomic analysis reveals that enterovirus F strain SWUN-AB001 infection activates JNK/SAPK and p38 MAPK signaling pathways in MDBK cells

**DOI:** 10.1186/s12917-018-1721-8

**Published:** 2018-12-13

**Authors:** Bin Zhang, Xinnuo Chen, Hua Yue, Wenqiang Ruan, Sinan Qin, Cheng Tang

**Affiliations:** 1 College of Life Science and Technology, Southwest Minzu University, No.16, South 4th Section 1st Ring Road, Chengdu, 610041 China; 2 Key laboratory of Ministry of Education and Sichuan Province for Qinghai-Tibetan Plateau Animal Genetic Resource Reservation and Utilization, Chengdu, 610041 China; 3 Animal Disease Prevention and Control Innovation Team in the Qinghai-Tibetan Plateau of State Ethnic Affairs Commission, Chengdu, 610041 China

**Keywords:** EV-F7, Transcriptomic analysis, DEG, JNK/SAPK, p38 MAPK

## Abstract

**Background:**

Enteroviruses (*Picornaviridae* family) have been widely detected in the feces from cattle with diarrhea. However, the mechanisms responsible for the pathogenicity of enteroviruses in cattle remain unclear. Recently, we isolated a novel EV-F7 strain called SWUN-AB001 from diarrheal yak (*Bos grunniens*) feces. To explore the pathogenic mechanisms of this novel virus, we used a transcriptomics approach to find genes with differential expression patterns in Madin-Darby bovine kidney (MDBK) cells during infection with SWUN-AB001 over time.

**Results:**

MDBK cells were sampled at 12 and 24 h post-infection (hpi) to represent the early and late stages of a SWUN-AB001 infection. Compared with the non-infected cells, 19 and 1050 differentially expressed genes (DEGs) were identified at 12 and 24 hpi, respectively. These DEGs were associated with disease, signal transduction, cellular process and cytokine signaling categories. At 24 hpi, the pathway enrichment analysis revealed that signal pathways such as c-Jun NH2-terminal kinase/ stress-activated protein kinase (JNK/SAPK) and mitogen-activated protein kinase (MAPK) pathways and cytokine-cytokine receptor interactions were associated with the interactions occurring between EV-F7 and MDBK cells. Our additional western blot analysis showed that the phosphorylation levels of JNK/SAPK and p38 MAPK proteins increased significantly in the MDBK cells at 24 hpi. The result indicated that infection with EV-F7 could activate JNK/SAPK and p38 MAPK pathways in MDBK cells, and possibly trigger large-scale cytokine production.

**Conclusion:**

Our transcriptome analysis provides useful initial data towards better understanding of the infection mechanisms used by EV-F7, while highlighting the potential molecular relationships occurring between the virus and the host’s cellular components.

**Electronic supplementary material:**

The online version of this article (10.1186/s12917-018-1721-8) contains supplementary material, which is available to authorized users.

## Background

*Enterovirus* (EV) genus (*Picornaviridae* family) members are small, non-enveloped icosahedral viruses with positive, single-stranded RNA genomes [1]. These viruses are widely distributed in humans and some other animal species as A-L groups, with *Rhinovirus* species belonging to groups A-C [1]. Approximately 50–80% of EV infections are asymptomatic or cause only mild, self-limiting illnesses [2–4]. However, some species can cause severe and potentially fatal infections. For instance, EV 71 (*Enterovirus A* species, serotype 71), a neurotropic virus, causes hand, foot, and mouth disease, and herpangina in children [5], while Coxsackievirus B3 (CV-B3; *Enterovirus B* species, serotype 3) the primary cause of viral myocarditis in humans, leads to cardiomyocyte death and life-endangering disease [6].

To aid their replication and survival, many EV species have evolved diverse strategies to evade the host’s innate immune responses [5, 7–11]. Infection with EV 71 can activate the c-Jun NH2-terminal kinase (JNK) and p38 mitogen-activated protein kinase (MAPK) signaling pathways, thereby contributing to increased viral replication and secretion of cytokines such as interleukin (IL)-2, IL-6, IL-10, and tumor necrosis factor (TNF)-α [8, 10]. Similarly, activation of both JNK and p38 MAPK pathways requires active replication of CV-B3, which likely contributes to viral progeny release [9]. Together, these studies indicate that the MAPK pathway plays important roles in the pathology of EV 71 and CV-B3 infections.

EVs were first identified in cattle in the late 1950s [12]. Originally two serotypes, BEV-1 and BEV-2 were described, with serotypes BEV-A and BEV-B being identified later on. BEV-A has since been renamed EV-E, while another serotype, BEV-F, has been renamed EV-F [13]. Currently, EV-E and EV-F species contain four (E1-E4) and seven (F1-F7) genotypes, respectively [13, 14]. Bovine EV infections are very common and viruses from this genus have been detected in cattle with severe enteric and respiratory diseases, as well as in the feces of presumably healthy animals [15–17]. Experimental infection trials using EV-E1 have failed to induce the clinical signs of disease, although the virus was detected in the intestinal tracts, brains, and hearts from the infected cattle [15]. Therefore, it remains unclear as to whether EV infection is a clinically relevant disease.

In our previous study, we isolated a novel EV-F7 strain (SWUN-AB001) from the feces of a yak (*Bos grunniens*) with severe diarrhea in the Qinghai-Tibetan Plateau [14]. The diarrheal fecal sample contained a higher prevalence of EV than the samples from healthy yaks in some regions, indicating that EV infections are potentially correlated with diarrhea in these animals [14]. Therefore, to explore the pathogenic mechanism of the novel SWUN-AB001 EV-F7 strain, we analyzed the transcriptomic profiles of infected Madin-Darby bovine kidney (MDBK) cells during the early and late infection periods with this strain. Our findings build on current knowledge about virus-host interactions and the molecular mechanisms of the cell signaling pathways that are activated during EV infections in yaks.

## Methods

### Cell culture and the SWUN-AB001 viral strain

MDBK cells (CCL-22, ATCC) were maintained in Dulbecco’s Modified Eagle’s Medium supplemented with 10% fetal bovine serum (Gibco) at 37 °C in a 5% CO_2−_enriched atmosphere. The novel EV-F7 SWUN-AB001 strain (TCID_50_ = 10^7.02^/mL) was originally isolated from the feces of a diarrheal yak in the Qinghai-Tibetan Plateau [14].

### Immunofluorescence assays

For the infection assays, the MDBK cells seeded in 24-well plates were incubated to 80% confluence. The cells were then infected with EV-F7 SWUN-AB001 at a multiplicity of infection (MOI) of 0.01, and re-incubated for 6, 12, 24 and 36 h. Mock infected cells were included as the controls.

As a measure of viral replication, an immunofluorescence assay was used to confirm the status of the viral infection at 6, 12, 24 and 36 h post-infection using a previously described method [11]. Briefly, the MDBK cells grown on Lab Tek chamber slides (Nunc) for 24 h were infected with SWUN-AB001 (MOI, 0.01) for 6, 12, 24 or 36 h, after which they were fixed with HistoChoice Clearing Agent (Sigma-Aldrich), permeabilized using 0.1% Triton X-100, and blocked with 1% bovine serum albumin. The cells were then incubated with an anti-viral antiserum prepared from EV-F7 strain SWUN-AB001 (1:100), followed by incubation with Alexa Fluor 488 anti-rabbit secondary antibody (1:1000) (Invitrogen). Nuclei acids were counterstained with 4′,6-diamidino-2-phenylindole (DAPI, Invitrogen). The chamber slides were then mounted onto glass slides using Fluorescence Mounting Medium (Dako), and the cells were observed and imaged using a fluorescence microscope (OLYMPUS IX73). All experiments were performed at least three times.

### cDNA library construction and sequencing

Cellular RNA was extracted from both infected (EV-12 h and EV-24 h) and non-infected (Con-12 h and Con-24 h) MDBK cells. Samples were collected in triplicate. Total RNA was isolated using TRIzol reagent (Life Technologies). For mRNA purification, the RNA samples were treated with DNase I, and poly (A) mRNA was purified using oligo-d (T) magnetic beads (Dynabeads). The purified mRNA was fragmented by divalent cation treatment at elevated temperature. The first-strand cDNA was transcribed from the cleaved RNA fragments using reverse transcriptase and random hexamer primers, followed by second-strand cDNA synthesis using DNA polymerase I and RNase H. The double-stranded cDNA was end-repaired using the Klenow fragment of T4 DNA polymerase and T4 polynucleotide kinase. A single adenine base was added via Klenow 3′–5′ exo-polymerase activity, followed by ligation to an adaptor or index adaptor using T4 DNA ligase. Adaptor-ligated fragments were separated and cDNA fragments of the correct size (200 ± 25 bp) were excised from an agarose gel. PCR was performed to selectively enrich and amplify the cDNA fragments.

Following validation on the Agilent 2100 Bioanalyzer and ABI StepOnePlus Real-Time PCR System, the resultant 12 libraries were sequenced using a single 50 bp end-read protocol using the BGISEQ-500 sequencing platform, as per the manufacturer’s instructions.

### Pre-processing of sequencing reads, and gene expression and differential gene analyses

Raw reads were subjected to a BGISEQ-500 quality control test using Soapnuke software (https://github.com/BGI-flexlab/SOAPnuke). Using the default parameters, we removed the “dirty” reads containing adaptor sequences, sequences where > 10% of the base calls were identified as unknown (“N”), and low quality reads. Only the remaining “clean” reads were used for the downstream analyses. The clean reads were aligned with the *Bos taurus* genome (*B. taurus* UMD 3.1.1, ftp://ftp.ncbi.nlm.nih.gov/genomes/Bos_taurus/) using Hisat2 (version 2.0.4) [18]. The *B. taurus* genome gene annotation (Bos UMD3.1 NCBI release) was also downloaded from the National Center for Biotechnology Information’s website. Following alignment, RNA-Seq by expectation maximization (or RSEM) [19] was used to normalize gene expression using the expected fragments per kilobase of transcript per million fragments sequenced (FPKM) method [20]. For the biological duplicate samples, NOIseq was used to calculate the log_2_ fold change (Log_2_FC) and probability for each gene in every comparison using strict criteria (Log_2_FC > 1 or Log_2_FC < − 1, probability > 0.7) [21]. Principal component analysis (PCA) can be used to reveal gene expression differences in biological samples based on the R language ggplot2 package, and this approach was used to analyze the sample data from the MDBK cells under the four different culture conditions (EV-12 h, Con-12 h, EV-24 h, and Con-24 h).

### Gene ontology (GO) and pathway enrichment analysis

GO analysis was used to determine the main biological functions of the DEGs. The hypergeometric test and false discovery rate (FDR) correction methods were used in the GO enrichment analysis to gain insights into the DEG functions. All of the GO annotation information was obtained from the Nr database, and we used GO::TermFinder (http://smd.stanford.edu/help/GO-TermFinder/GO_TermFinder_help. shtml) to obtain information about the gene classes. In all cases, *p* < 0.05 was considered to be statistically significant. Statistical analyses relating to the hypergeometric test and the FDR method were conducted using the R package, and all the GO analyses used a custom-made perl script. Pathway enrichment analysis was performed using KAAS (KEGG Automatic Annotation Server, http://www.genome.jp/tools/kaas/) to functionally annotate the genes using BLAST comparisons against the manually curated KEGG database. The threshold of significance was defined as *p* < 0.05.

### Western blot analysis

MDBK cells were seeded in 24-well plates and incubated to 80% confluence. The cells were infected with the EV-F7 SWUN-AB001 strain (MOI, 0.01) and incubated for a further 12 h or 24 h. Western blot analysis was then performed as previously described [11], using polyclonal antibodies against JNK/SAPK, phospho-JNK/SAPK, p38 MAPK, phospho-p38 MAPK, or GADPH (Cell Signaling Technology). Horse radish peroxidase-conjugated goat anti-mouse and goat anti-rabbit IgG antibodies were obtained from Abbkine. Densitometry values for the immunoblot signals were obtained from three separate experiments using FusionCapt Advance software (Vilber Lourmat).

### Quantitative real-time polymerase chain reaction (qRT-PCR) verification of the BGI-500 sequencing data

DEGs were examined by qRT-PCR to confirm the accuracy of the sequencing data. The primer sequences for the eight selected DEGs are listed in Additional file 1: Table S1. The total RNA extracted from the infected and non-infected MDBK cells at 12 h and 24 h using TRIzol reagent (Life Technologies) was reverse transcribed using the SuperScript III First-Strand Synthesis System (Life Technology). For the qRT-PCR analysis, the SYBR Premix Ex Taq II (Tli RNaseH Plus) Kit (TakaRa) and the ABI 7500 FAST real-time PCR system (ABI) were used according to each manufacturer’s instructions. The relative expression level of each gene was calculated using the 2^−ΔΔCt^ method.

## Results

### Viral infection

**Fig. 1:**
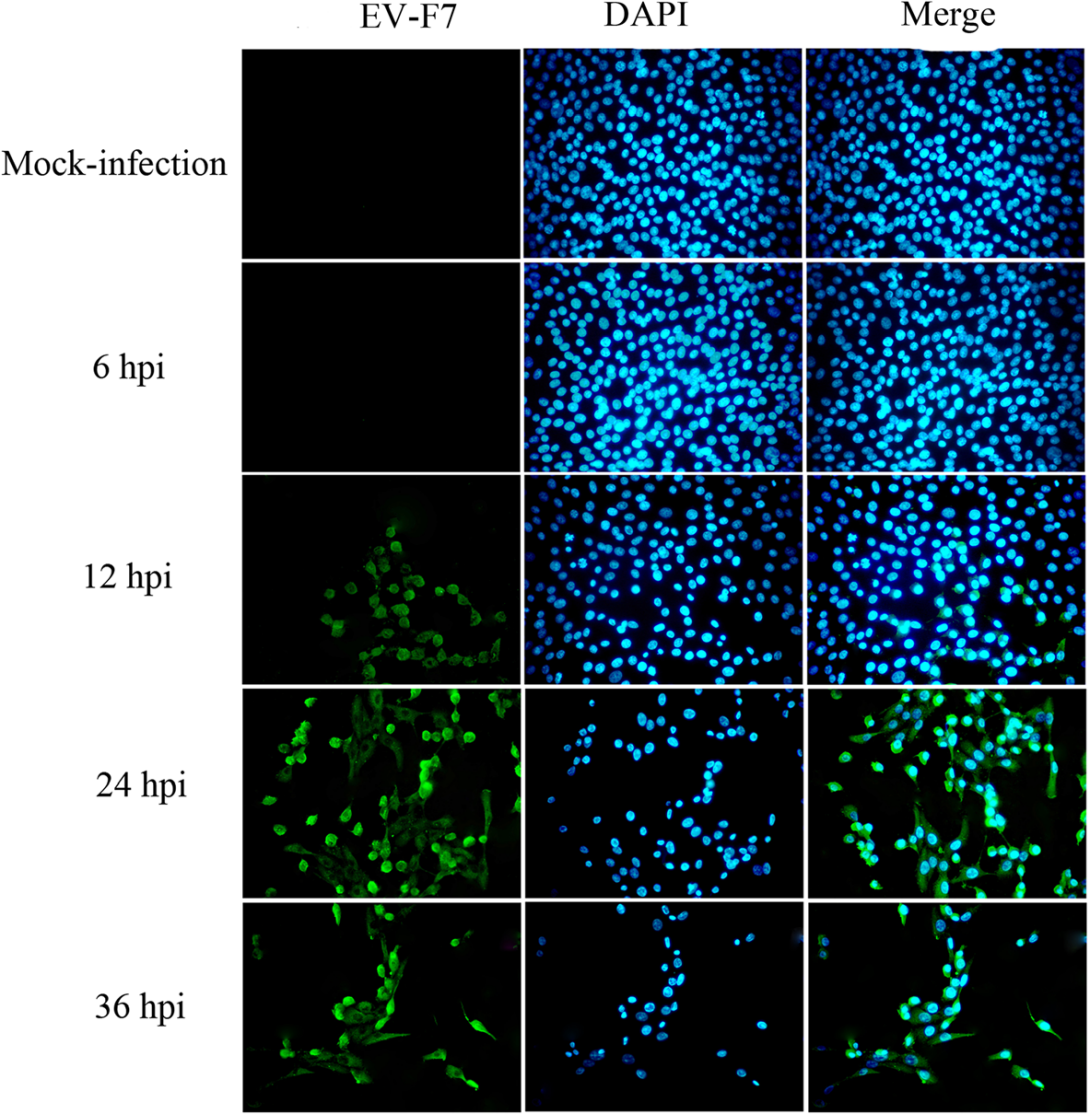
Immunofluorescence detection of EV-F7 infection in MDBK cells at 6, 12, 24 and 36 h post-infection

To identify the early and late viral infection periods, MDBK cells were infected with EV-F7 SWUN-AB001 (MOI, 0.01) for 6, 12, 24 or 36 h, and then examined using an immunofluorescence assay. Viral particles were observed at 12 h post-infection (hpi), at which point about 20% of the cells were positive for EV-F7 but lacked any obvious cytopathic effects (CPEs) (Fig. 1). The result was consistent with how the early part of an infection with this virus proceeds. A significant increase in the number of viral particles was observed at 24 hpi, and this was associated with an obvious decrease in the number of viable cells, indicating the onset of CPEs. At 36 hpi, the numbers of both viable cells and viral particles had significantly decreased. Therefore, the 12 and 24 hpi time points were selected to represent the early and late periods of infection with the SWUN-AB001 strain in MDBK cells, respectively.

### Transcriptome sequencing and gene expression

To investigate the changes occurring in the cellular components during the early and late expression periods of the EV-F7 infection, we used RNA-Seq to analyze the transcription levels in the MDBK-infected cells. The sequencing libraries were prepared in triplicate from the MDBK cells under four different culture conditions (EV-12 h, Con-12 h, EV-24 h, and Con-24 h), which were sequenced using BGI-500.

After removing the low-quality reads, we obtained an average of 23,950,659 and 23,953,122 clean reads from the EV-12 h and EV-24 h libraries, respectively, and an average of 23,947,049 and 23,953,282 clean reads from the Con-12 h and Con-24 h libraries, respectively. The proportion of clean reads in all the samples was greater than 99%, thus demonstrating the reliability of the sequencing data quality (Table 1). Alignment analysis of the sequences from the infected and control samples collected at 12 hpi showed that 78.88 and 83.32%, respectively, mapped to the *B. taurus* genome. Of the reads from the 24 hpi samples, 55.76% of those from the infected samples and 82.67% of those from the control samples mapped to the *B. taurus* genome. Thus, the serious CPE observed at 24 hpi likely affected the amount of data obtained from the MDBK cells at this time point.

**Table 1:** Summary of the sequencing reads from the MDBK cells with and without EV-F7 infection

Sample name	MDBK cells (NO.)	Clean Data (bp)	Clean Reads Number	Clean Rate (%)	Genome Mapping (%)	Detected Gene NO.
EV-12 h	1	1,197,656,100	23,953,122	99.98	80.08	18,658
	2	1,197,653,000	23,953,060	99.98	79.71	18,659
	3	1,197,532,950	23,950,659	99.97	75.88	18,681
Con-12 h	1	1,197,568,750	23,951,375	99.97	82.73	18,794
	2	1,197,511,250	23,950,225	99.97	83.32	18,802
	3	1,197,352,450	23,947,049	99.95	82.67	18,832
EV-24 h	1	1,197,609,800	23,952,196	99.97	55.76	17,918
	2	1,197,651,050	23,953,021	99.98	57.68	17,995
	3	1,197,636,250	23,952,725	99.98	60.44	18,109
Con-24 h	1	1,197,637,200	23,952,744	99.98	82.38	18,848
	2	1,197,664,100	23,953,282	99.98	82.67	18,818
	3	1,197,565,550	23,951,311	99.97	82.65	18,820

Gene expression levels were measured by short-read mapping and are presented in reads per kilobase per million mapped reads (RPKM), adjusted by a normalization factor. In total, we detected 21,317 expressed genes or transcripts in all 12 samples, and within each sample 17,918 to 18,848 expressed genes or transcripts were detected, respectively (Table 1, Additional file 2: Table S2). According to the transcriptome features, the standard uniquely mapped reads approach we used was adjusted by constructing the sequence clusters prior to read mapping. The PCA results revealed that the transcriptome profiles of the samples that were subjected to the same culture conditions were clustered together (Additional file 3: Figure S1), which confirms the reproducibility of the transcriptomic sequencing at the different time points we analyzed.

### DEG identification in the MDBK cells

We next aimed to identify the DEGs in the MDBK cells under different culture conditions. A gene was deemed to be differentially expressed if the fold change of the FPKM expression values was at least two, and the divergence probability was at least 0.7. Using both comparisons for each library pair (Con-12 h vs EV-12 h, and Con-24 h vs EV-24 h), 19 and 1050 genes were identified as being differentially expressed at the 12 h and 24 h time points, respectively. Of the 19 DEGs identified at 12 hpi, 12 were up-regulated and seven were down-regulated (Additional file 4: Table S3). A smaller number of DEGs were identified at the 12 h time point probably relates to the fact that 99% of the MDBK cells did not become infected with SWUN-AB001 at a MOI of 0.01, and the effects of the viral infection on the host cells were, therefore, quite limited. Of the 1050 DEGs identified at 24 hpi, 103 were up-regulated and 947 were down-regulated (Additional file 4: Table S3). Fifty-three genes with unknown functions were identified, including 46 significantly down-regulated and seven up-regulated genes. In addition, 216 non-coding (nc) RNAs were identified among the down-regulated DEGs at 24 hpi. ncRNAs are important functional RNA molecules that play critical roles during almost every viral infection process, including regulating viral growth, replication and cell death [22, 23]. In the case of an EV 71 infection, virus-induced cellular ncRNAs are known to modulate the cellular and infection processes and contribute to pathogenesis by targeting either host mRNAs or virus RNAs [24]. Accumulating evidence supports the importance of ncRNAs in EV 71 infection-related pathogenesis, and these molecules might be a viable target of anti-virus strategies [25, 26]. Therefore, we speculated that the numerous down-regulated ncRNAs might play roles in the EV-F7 infection-related pathogenesis or induce a range of anti-virus responses in the host cells. Hence, it is likely that the roles played by ncRNAs in EV-F7 infections will attract further study.

As illustrated by the Venn-diagram in Additional file 5: Figure S2, 12 DEGs overlapped the 12 h and 24 h time points. Of these, 10 genes were up-regulated and two were down-regulated (Additional file 5: Figure S2). The expression level of *TNFAIP3* (A20), which codes for TNF-α-induced protein 3, was found to have increased at both time points, an interesting finding considering the critical role of TNFAIP3 in terminating NF-κB pathway activation [27]. Early growth response 1 (*EGR1)* mRNA levels were also found to have significantly increased at both 12 and 24 hpi. EGR1, a multifunctional transcription factor, regulates diverse biological functions, including inflammation, apoptosis, differentiation, tumorigenesis, and even viral infections [28]. It is possible that infection with EV71 activates *EGR1* expression to enhance viral replication [29]; if so, this suggests that *EGR1* possibly plays a role in EV-F7 infections.

### Functional analysis and biological enrichment of DEGs

**Fig. 2:**
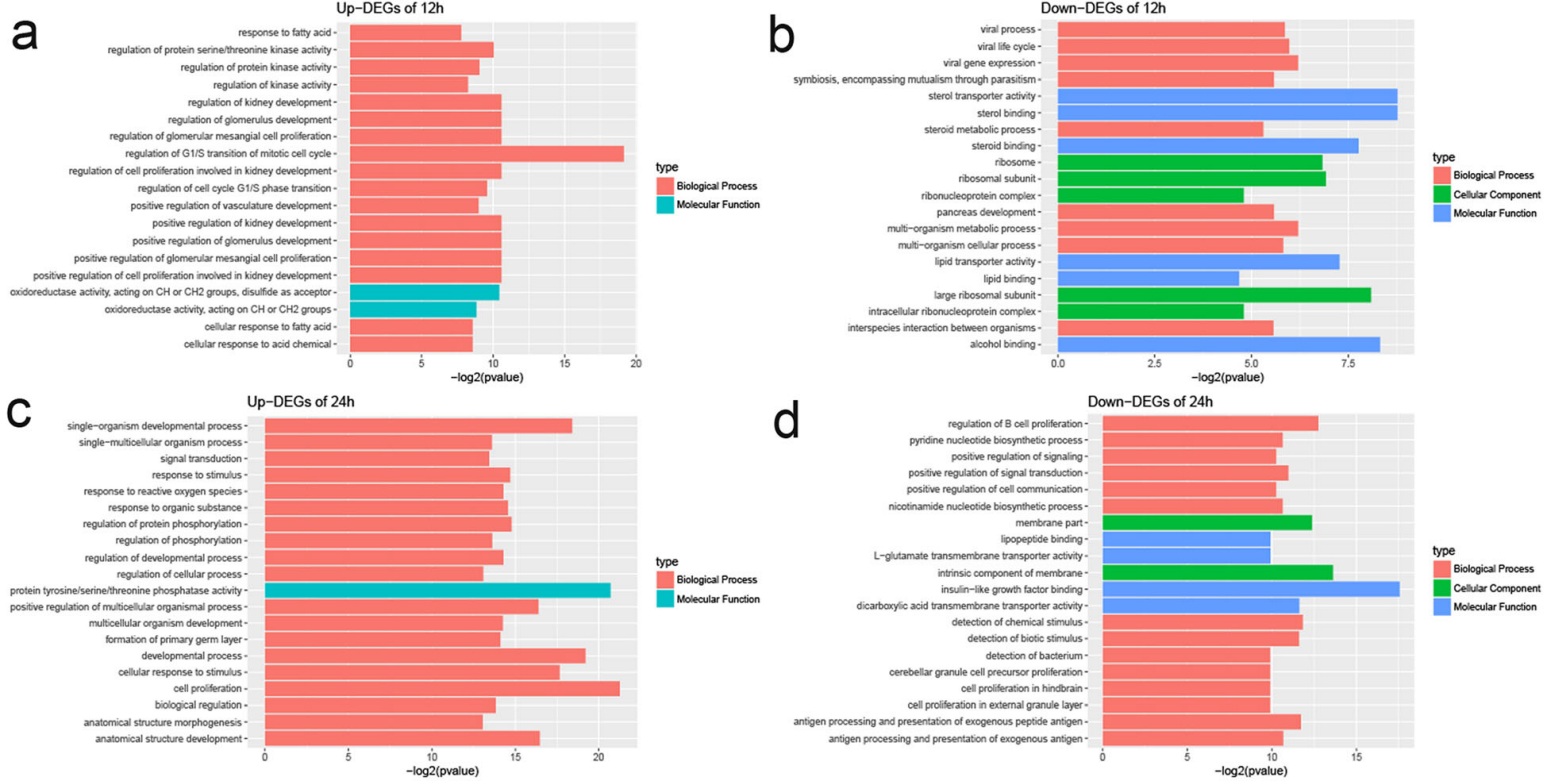
GO assignments for DEGs in MDBK cells following infection with EV-F7. GO categories are shown for up-regulated genes at 12 (**a**) and 24 h post-infection (hpi) (**c**), and for down-regulated genes at 12 (**b**) and 24 hpi (**d**). The GO categories included biological processes, cellular components, and molecular functions. The x-axis shows the GO categories and the y-axis shows the number of genes

To gain insight into the biological roles of the DEGs in our study, we performed a gene ontology (GO) enrichment analysis on each of the genes. The GO analysis revealed that the DEGs were enriched in many GO categories, including biological processes, cellular components, and molecular function (Additional file 6: Table S4). At 12 hpi, four up-regulated genes (*RRM2*, *EGR1*, *SPRY4*, and *PIM1*) had GO annotations (*p* < 0.05) (Fig. 2a), such as ‘regulation of cell cycle G1/S phase transition’ (GO:1902806), ‘regulation of protein kinase activity’ (GO:0043549) and ‘regulation of transferase activity’ (GO:0051338), while the only down-regulated gene (*STARD3*) is listed as having ‘sterol transporter activity’ (GO:0015248) and ‘lipid transporter activity’ (GO:0005319) (Fig. 2b). At 24 hpi, there was an increase in the number of DEGs in the host cells and they were found to be involved in multiple biological processes. Among the up-regulated genes, 57, 58 and 61 were mapped to ‘cellular component’, ‘molecular function’ and ‘biological process’, respectively (*p* < 0.05) (Fig. 2c and d). The GO terms relating to the biological processes of genes up-regulated at 24 hpi included ‘single-organism cellular process’ (GO: 0044763), ‘biological regulation’ (GO: 0065007) and ‘response to stimulus’ (GO: 0050896). However, 374 down-regulated genes at the 24 hpi time point were identified as being enriched in GO terms like ‘insulin-like growth factor binding’ (GO: 0005520) and ‘intrinsic component of membrane’ (GO: 0031224). In addition, we found 4, 6, 12 and 32 of the genes down-regulated 24 hpi were associated with ‘adaptive immune response’ (GO: 0002250), ‘regulation of B cell proliferation’ (GO: 0030888), ‘positive regulation of signaling’ (GO: 0009967) and ‘immune system process’ (GO: 0002376), respectively, (*p* < 0.05).

**Fig. 3:**
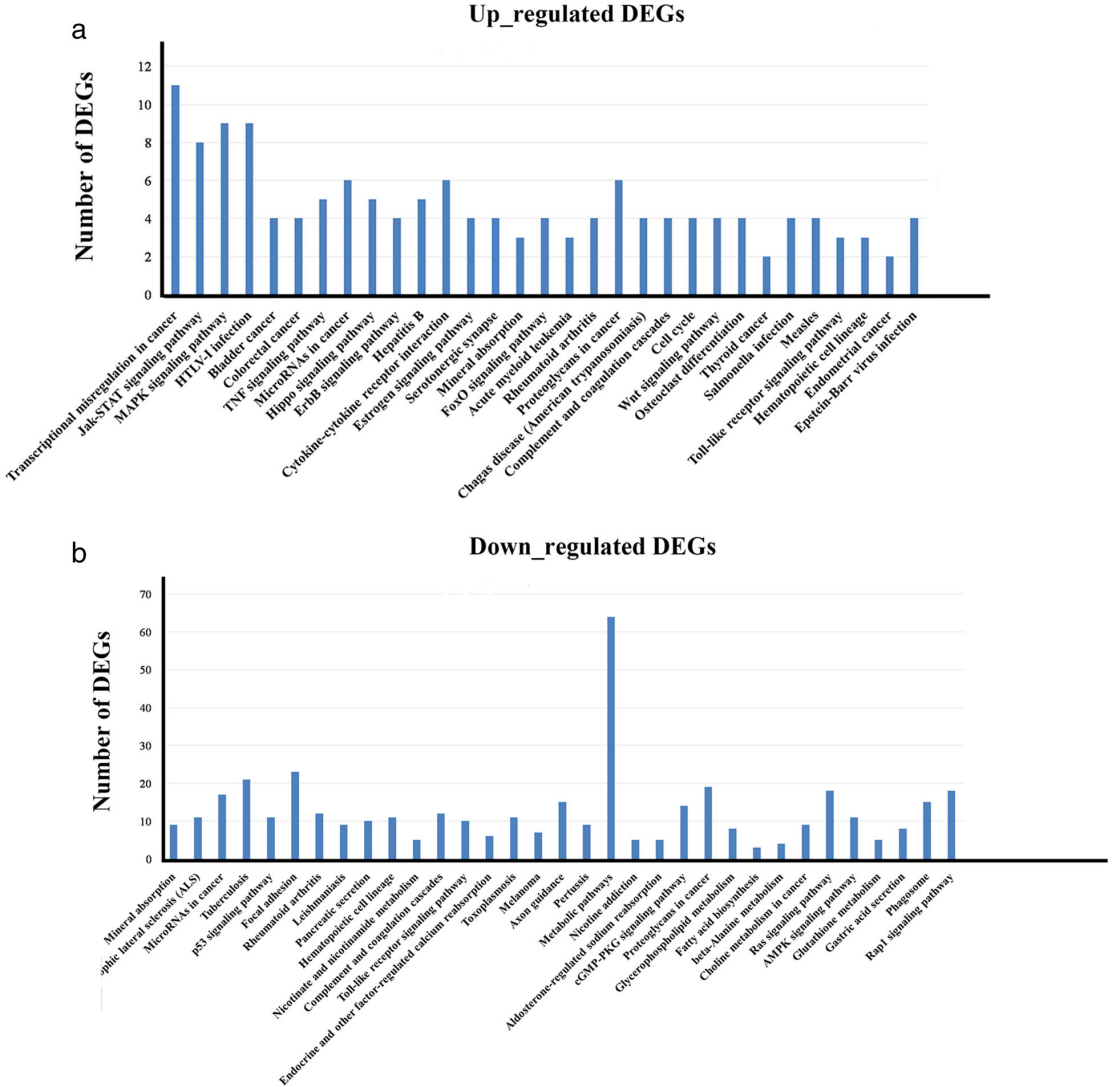
KEGG classification of the DEGs at 24 h post EV-F7 infection. The KEGG classifications for the up-regulated (**a**) and down-regulated (**b**) genes are shown. The x-axis indicates the pathway and the y-axis indicates the number of DEGs

KEGG analysis of the DEGs assigned only four and two KEGG pathways to the up- and down-regulated DEGs at 12 hpi, respectively. At 24 hpi, 103 of the up-regulated genes were significantly enriched in 31 pathways that were mainly related to disease, signal transduction, and cytokine signaling (*p <* 0.05) (Fig. 3, Additional file 7: Table S5). Nine DEGs showed MAPK signaling pathway enrichment; this pathway can activate signaling cascades to produce inflammation and mediate viral replication. These DEGs, which include *c-fos*, *c-Myc*, *c-Jun*, *DUSP1* and *HSP70*, are reportedly involved in initiating the MAPK signaling pathway [30]. Host MAPK signaling pathway activation by viruses triggers the ERK1/2, JNK, and p38 MAPK signaling pathways, thereby contributing to inflammatory cytokine secretion, induction of apoptosis in the infected cells, and enhanced viral replication [30]. The DEGs involved in cytokine signaling were also up-regulated following infection with EV-F7, and included *IL-6*, *IL-11*, leukemia inhibitory factor (*LIF*), granulocyte-macrophage colony-stimulating (*GM-CSF*), and colony stimulating factor 2 (*CSF2*). EV infections often induce high levels of inflammatory cytokines in host cells, resulting in a cytokine storm, as has been observed with EV 71 and CV-B4 infections [8, 31].

In this study, the 947 down-regulated genes were significantly enriched in 33 signaling pathways and were mainly associated with disease, cellular processes, and signal transduction (*p* < 0.05), such as the p53 signaling pathway (Fig. 3; Additional file 7: Table S5). The p53 signaling pathway has been implicated in a large number of biological processes, including cell growth arrest and apoptosis in response to DNA damage [32]. Many viruses, including CV-B3 and poliovirus [33, 34], have evolved strategies to inhibit p53 surveillance pathways and prevent early apoptosis, allowing for effective viral replication. In this study, the KEGG analysis revealed that the p53 signaling pathway was significantly inhibited in the MDBK cells at 24 hpi, suggesting that EV-F7 interferes with this signaling pathway.

### SWUN-AB001 activates JNK/SAPK and p38 MAPK signaling pathways in infected MDBK cells

**Fig. 4:**
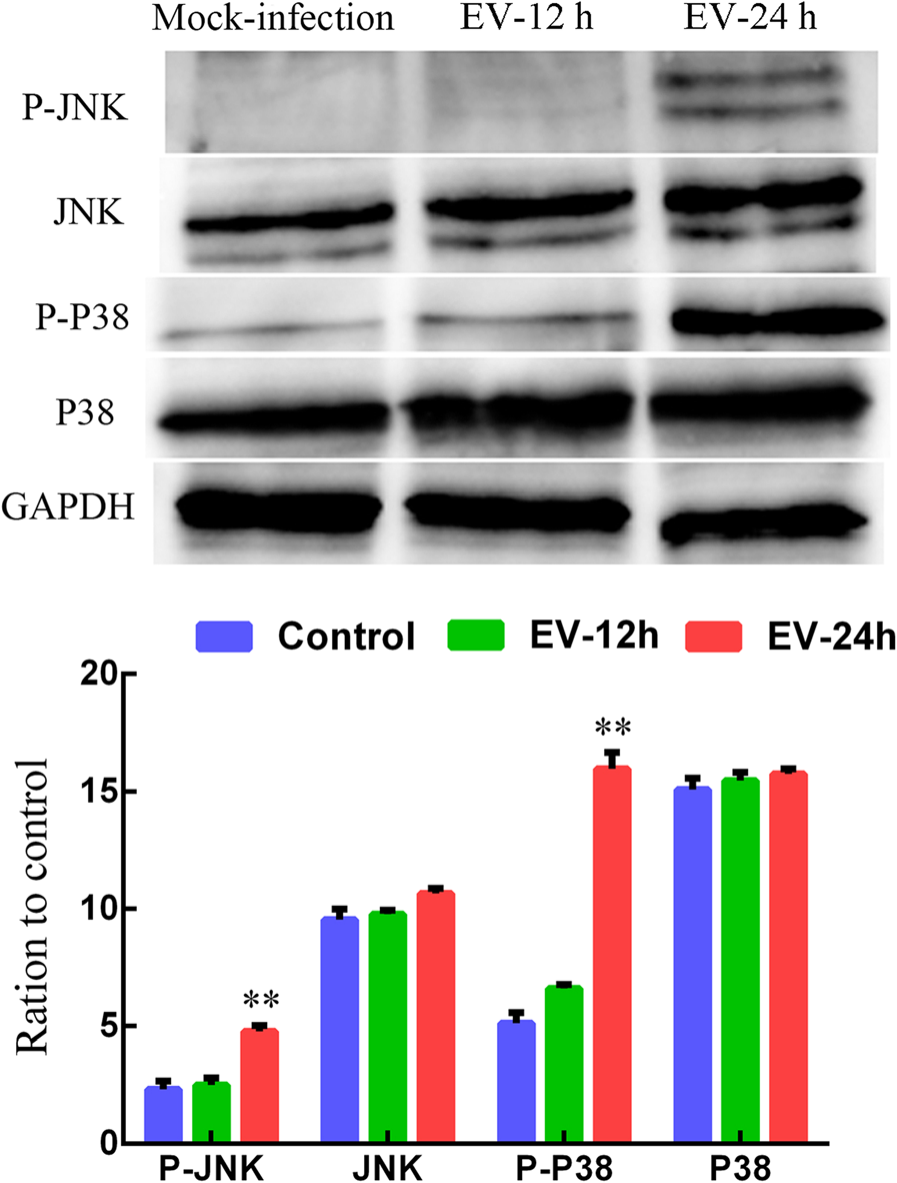
EV-F7 infection activated MAPK pathways in the MDBK cells. The MDBK cells were stimulated with EV-F7 at a MOI of 0.01 for 12 or 24 h. Western blotting of p38 MAPK, phospho-p38 MAPK, JNK/SAPK, phospho-JNK/SAPK, and GAPDH was performed. Bar charts show the relative protein expression levels quantified from three separate experiments. The values presented are the mean values ± standard deviations, and the data were analyzed using a one-way analysis of variance

The transcriptomic results suggest that the MAPK signaling pathway is involved in infection with EV-F7. To assess whether this pathway was activated in EV-F7-infected MDBK cells, the total and phosphorylated amounts of JNK/SAPK and p38 MAPK at 12 and 24 hpi were measured by western blotting, with unstimulated cells used as the mock-stimulated control. MDBK cells were infected with EV-F7 at an MOI of 0.01 and then incubated for 12 h or 24 h. The EV-F7 infection had no obvious effect on the total or phosphorylated amounts of JNK/SAPK and p38 MAPK at 12 hpi, but the amounts of phosphorylated JNK/SAPK and p38 were seen to significantly increase at 24 hpi (Fig. 4). These results indicate that phosphorylation of JNK/SAPK and p38 MAPK might play an important role in EV-F7 replication.

### Transcriptome data verification by qRT-PCR

**Fig. 5:**
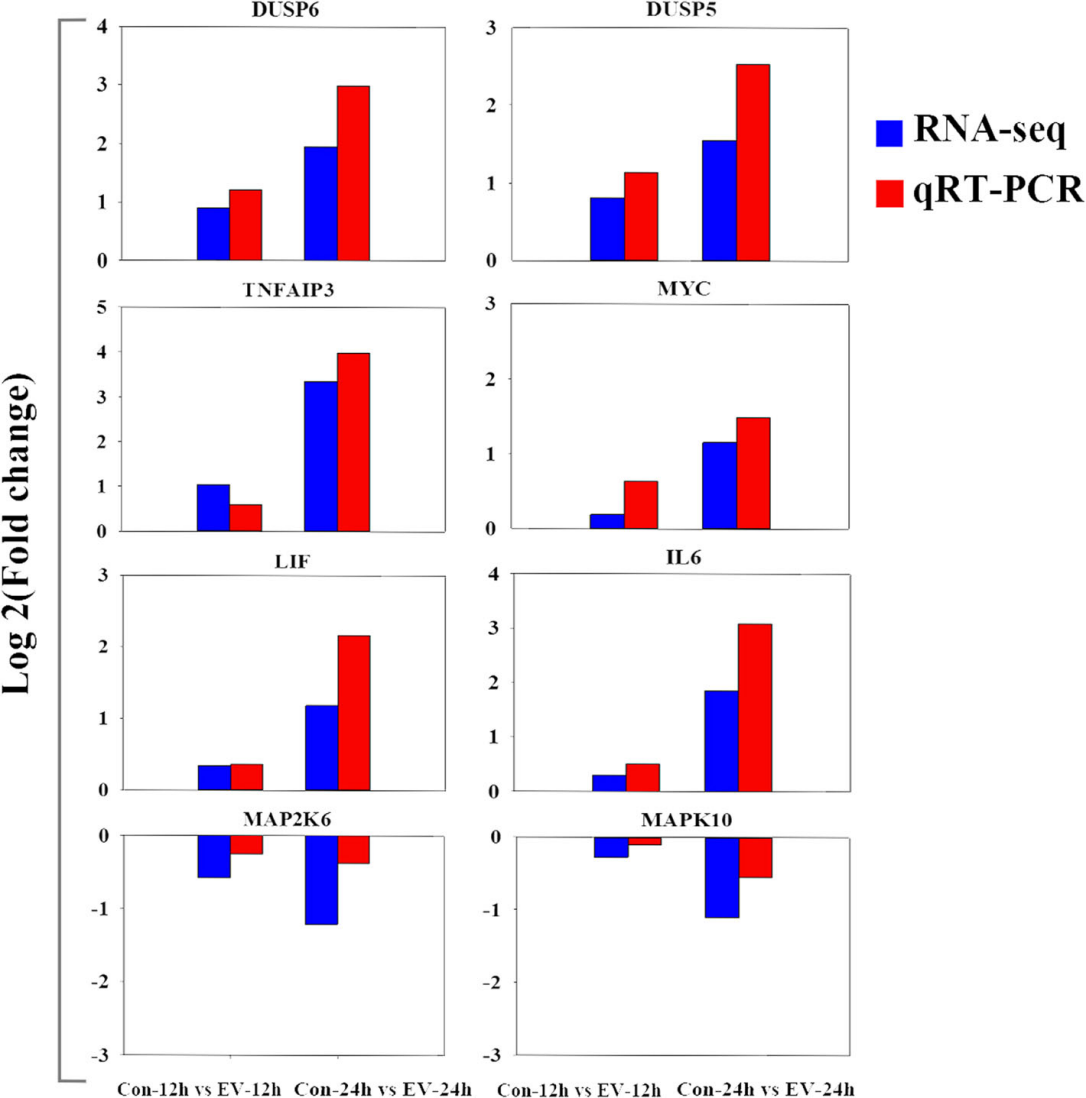
Validation of the expression patterns of six differentially expressed genes by qRT-PCR analysis. The log_2_ (fold change) values derived from the RNA-seq analysis of eight genes were compared with those obtained by qRT-PCR

To further evaluate our DEG library, six up-regulated and two down-regulated DEGs were selected for qRT-PCR analysis. Seven housekeeping genes (*β-actin*, *GAPDH*, *B2M*, *BLM*, *TBP*, *SDHA*, and *BLM*), which were selected from the RNA-Seq data, were tested as potential internal reference genes. The results from this analysis showed that only *B2M* was stably expressed across the different time points in both the control and the infection group (Additional file 2: Table S2). *B2M* was, therefore, selected as the internal reference for the qRT-PCR experiments, the results of which showed the same patterns of expression as those observed with the DEG data, which confirms the validity of the sequencing results (Fig. 5).

## Discussion

The novel EV-F7 SWUN-AB001 strain was previously isolated from the feces of a diarrheal yak [14]. In the present study, RNA-Seq analysis of EV-F7-infected MDBK cells was conducted to identify the genes involved in viral infection mechanisms, immune response evasion, and cell signaling pathway activation. We first confirmed that the initial period of infection occurred at 12 hpi using immunofluorescence assays that showed the presence of viral particles in the MDBK cells in the absence of any CPEs. At 24 hpi, CPE was evident in the MDBK cells, with an obvious decrease in the number of viable cells, suggesting that this was the late infection stage. The early and late infection periods are important stages for viral replication, and identifying genes with differential expression patterns in host cells during these periods should augment current understanding about the mechanisms involved in viral infections. Therefore, high-throughput mRNA sequencing of infected MDBK cells at 12 and 24 hpi was used to identify DEGs.

During the early period of EV-F7 infection of MDBK cells, the virus-infected host cells triggered the host’s innate immune signaling pathways. The transcriptome analysis revealed that 19 DEGs, including 12 that were up-regulated and seven that were down-regulated, were identified at 12 hpi. The DEGs involved in viral replication included *TNFAIP3*, *EGR1*, and *Pim-1*. TNFAIP3, which is also called A20, is a cytoplasmic protein that plays a key role in negatively regulating the inflammatory response and the NF-κB signaling pathway [24]. Some viruses such as CV-B3, influenza virus and bovine viral diarrhea virus can induce A20 expression during their infections [35–37]. In the present study, *TNFAIP3* expression levels increased significantly at both 12 and 24 hpi in the MDBK cells, a result supported by our independent qRT-PCR analysis. Genes in the NF-κB signaling pathway were not identified as being differentially expressed by KEGG analysis in the current study. Therefore, we speculate that TNFAIP3 might participate in inhibiting the NF-κB signaling during the early and late infection stages of an EV-F7 infection, implying that it might also regulate innate immune signaling or be involved in antivirus responses to infection with EV-F7. *EGR1* expression is associated with many different viral infection types, including EV71, HIV, herpes simplex virus-1, and Kaposi’s sarcoma-associated herpesvirus [29]. EGR1 activates microRNA-141 expression and suppresses production of eukaryotic initiation factor 4E, thereby disrupting host protein synthesis and promoting replication of EV71 [38]. Pim1 is a constitutively active serine/threonine kinase known to be involved in cell survival in that it increases the threshold for apoptosis. Inhibition of Pim1 kinase activity in human rhinovirus-16 infected primary bronchial epithelial cells was found to enhance the onset of cell death, resulting in reduced viral replication [39]. In the current study, both *EGR1* and *Pim1* were significantly up-regulated in MDBK cells at 12 and 24 hpi and might, therefore, be involved in viral replication.

At 24 h post EV-F7 infection, 1050 DEGs, including 103 that were up-regulated and 947 that were down-regulated were identified. These genes are involved in the pathways associated with disease, signal transduction, cytokine signaling, and cellular processes, all of which might be involved in viral pathogenesis. Several of the up-regulated DEGs we identified at 24 hpi were proinflammatory cytokines and chemokines (e.g., *IL-6*, *IL-11*, *LIF*, *GM-CSF* and *CSF2*), and the mRNA expression levels of *IL-6* and *LIF* were confirmed to be accurate by qRT-PCR analysis. These results suggest that the release of cytokines and chemokines contributes to EV-F7 infection in MDBK cells. Cytokines and chemokines are usually induced by oxidant stress and viral infection, and they can cause host cell damage, chronic inflammation, and other immune responses [40]. EV71 infection induces high levels of inflammatory cytokines in host cells such as IL-6, IL-10 and TNF-α, with a cytokine storm recognized as the main cause of severe cardiopulmonary manifestations during this infection [8]. In addition, CV-B4 infections, which are mainly associated with meningoencephalitis, neonatal myocarditis and type-1 diabetes, can also promote cytokine and chemokine production in host cells (e.g., IL-6, LIF, and GM-CSF) [31]. Therefore, we speculate that the pathogenicity of EV-F7 might be partly the result of excessive induction of harmful proinflammatory responses and, if correct, this would represent a significant leap forward in our understanding of the viral pathogenesis of EV-F7 infections.

The family of serine/threonine protein kinases known as MAPKs are widely conserved among eukaryotes, and are involved in many cellular processes, including inflammation, proliferation, differentiation, movement, and cell death [8]. MAPK pathways are required for inflammation, infection, and replication of EV during infection, specifically the JNK/SAPK and p38 MAPK pathways [30]. The use JNK or P38 MAPK inhibitors during an EV71 infection has been found to severely inhibit the induction of cytokines and viral replication in the host cells, indicating that both signaling pathways are beneficial to EV71 infections, and can positively regulate the secretion of inflammatory cytokines in host cells [8, 41]. In the current study, the MAPK signaling pathways were enriched in the up-regulated DEGs, suggesting that they are involved in viral replication and host inflammation during EV-F7 infection of MDBK cells. To further investigate the potential mechanism, we examined the JNK/SAPK and P38 MAPK signaling pathways during EV-F7 infection of MDBK cells. The results showed that phosphorylation of the JNK/SAPK and P38 MAPK proteins increased significantly at 24 hpi, indicating that these pathways play important roles in infection with EV-F7.

## Conclusions

In this study, we extensively characterized the transcriptome of MDBK cells during the early and late stages of infection with EV-F7. A great number of the genes found to be differentially expressed upon infection with EV-F7 in MDBK cells, and were functionally annotated, were associated with disease, signal transduction, cellular process and cytokine signaling. Our findings provide a rational basis for conducting additional research aimed at unraveling the mechanisms underlying the infectious nature of this novel EV-F strain from yak.

## Additional files

Additional file 1: **Table S1.** Sequences of the PCR primers used in this study. (DOCX 16 kb)

Additional file 2: **Table S2.** List of all the genes expressed in the MDBK cells. (XLSX 4835 kb)

Additional file 3: **Figure S1.** Principal component analysis of the four treatment groups for MDBK cells (three biological replicates for each treatment). (JPG 328 kb)

Additional file 4: **Table S3.** List of the differentially expressed genes in the MDBK cells following infection with EV-F7 at 12 and 24 h post-infection. (XLSX 290 kb)

Additional file 5: **Figure S2.** Venn diagram of the up- and down-regulated genes identified following comparisons of the Con-12 h vs. EV-12 h and Con-24 h vs. EV-24 h treatment groups. (JPG 287 kb)

Additional file 6: **Table S4.** Enrichment analysis of the biological processes GO terms assigned to DEGs in the MDBK cells 12 and 24 h post infection with EV-F7. (XLSX 249 kb)

Additional file 7: **Table S5.** KEGG pathway analysis of the differentially expressed genes in MDBK cells following EV-F7 infection at 12 and 24 h post-infection. (XLSX 61 kb)

## Abbreviations

BEV: Bovine enterovirus
CPEs: Cytopathic effects
CSF2: Colony stimulating factor 2
CV-B3: Coxsackievirus B3
CV-B4: Coxsackievirus B4
DEGs: Differentially expressed genes
EGR1: Early growth response-1
EV: *Enterovirus*
EV-E: Enterovirus E
EV-F: Enterovirus F
FDR: False discovery rate
FPKM: Expected fragments per kilobase of transcript per million fragments sequenced
GM-CSF: Granulocyte-macrophage colony-stimulating factor
GO: Gene ontology
hpi: Hours post-infection
IL: Interleukin
JNK/STAT: c-Jun NH2-terminal kinase/ stress-activated protein kinase
LIF: Leukemia inhibitory factor
Log_2_FC: log_2_ fold change
MAPK: Mitogen-activated protein kinase
MDBK: Madin-Darby bovine kidney
MOI: Multiplicity of infection
ncRNA: Non-coding RNA
PCA: Principal component analysis
qRT-PCR: Quantitative real-time polymerase chain reaction
TNF: Tumor necrosis factor
