# Supplementary material for: Transcriptomic analysis reveals that enterovirus F strain SWUN-AB001 infection activates JNK/SAPK and p38 MAPK signaling pathways in MDBK cells
Source: BMC Vet Res. 2018 Dec 13;14:395. doi: 10.1186/s12917-018-1721-8 (PMC6293526; doi:10.1186/s12917-018-1721-8)
Supplement: Supplementary file 1 — Table S1. Sequences of the PCR primers used in this study. (DOCX 16 kb) [file 12917_2018_1721_MOESM1_ESM.docx]

**Table S1.** Sequences of the PCR primers used in this study.

| Primers | Nucleotide sequence (5'–3') | PCR product length (bp) |
| --- | --- | --- |
| LIF | F- AGGTCTTGGCGGCAGGAGT | 102 |
|  | R- TGGCACAGGTGGCGTTGA |  |
| IL-6 | F-TGAGTCTGAAAGCAGCAAGGA | 138 |
|  | R-TACTCCAGAAGACCAGCAGTGG |  |
| TNFAIP3 | F- TTTGAACTTGTCCAGCACG | 223 |
|  | R- TTGGGACTTTCGTTTGGT |  |
| DUSP5 | F-AGTGCGAGTTCCTTGCCA | 192 |
|  | R- GGACCTTGCCTCCCTTTT |  |
| DUSP6 | F- GCAGCGACTGGAACGAGA | 186 |
|  | R- ACTGGCAACGGTGGCGAG |  |
| MYC | F- CCCTACCCGCTCAACGACA | 232 |
|  | R- GGGGCTGCCTCTTTTCCA |  |
| MAP2K6 | F- GCGACTTCGGAATCAGCG | 207 |
|  | R- GGAAAGGAGTCCCCCACG |  |
| MAPK10 | F- ACGCTTGGGTTTTCTTGG | 204 |
|  | R- TGGTGTTGCCGTTGGTGT |  |
| B2M | F- TCCAGCGTCCTCCAAAGATT | 135 |
|  | R- TCTTCTCCCCATTCTTCAGCAA |  |
