# Supplementary figures and images for: Transcriptomic analysis reveals that enterovirus F strain SWUN-AB001 infection activates JNK/SAPK and p38 MAPK signaling pathways in MDBK cells
Source: BMC Vet Res. 2018 Dec 13;14:395. doi: 10.1186/s12917-018-1721-8 (PMC6293526; doi:10.1186/s12917-018-1721-8)

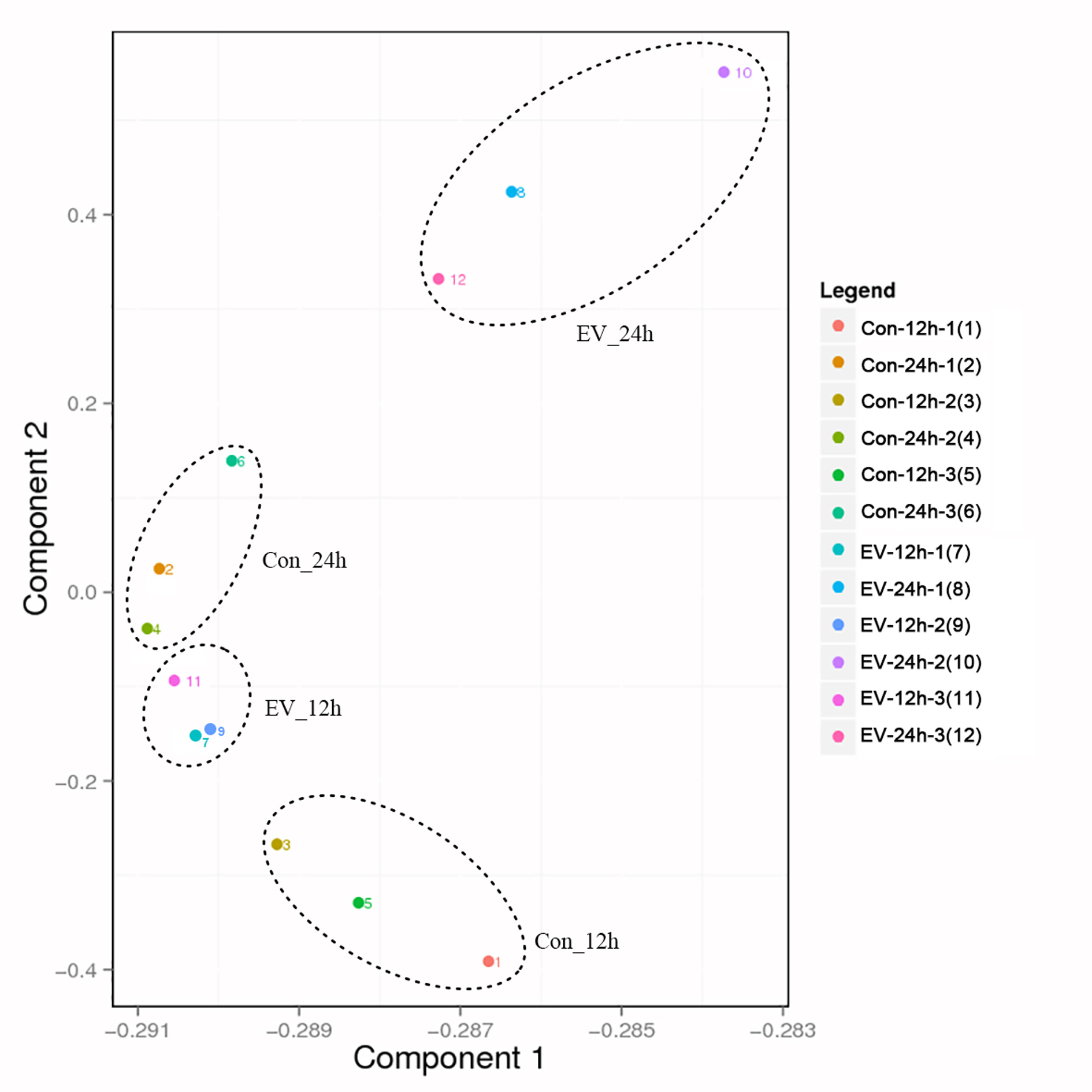

Supplement: Supplementary file 3 — Figure S1. Principal component analysis of the four treatment groups for MDBK cells (three biological replicates for each treatment). (JPG 328 kb) [file 12917_2018_1721_MOESM3_ESM.jpg]

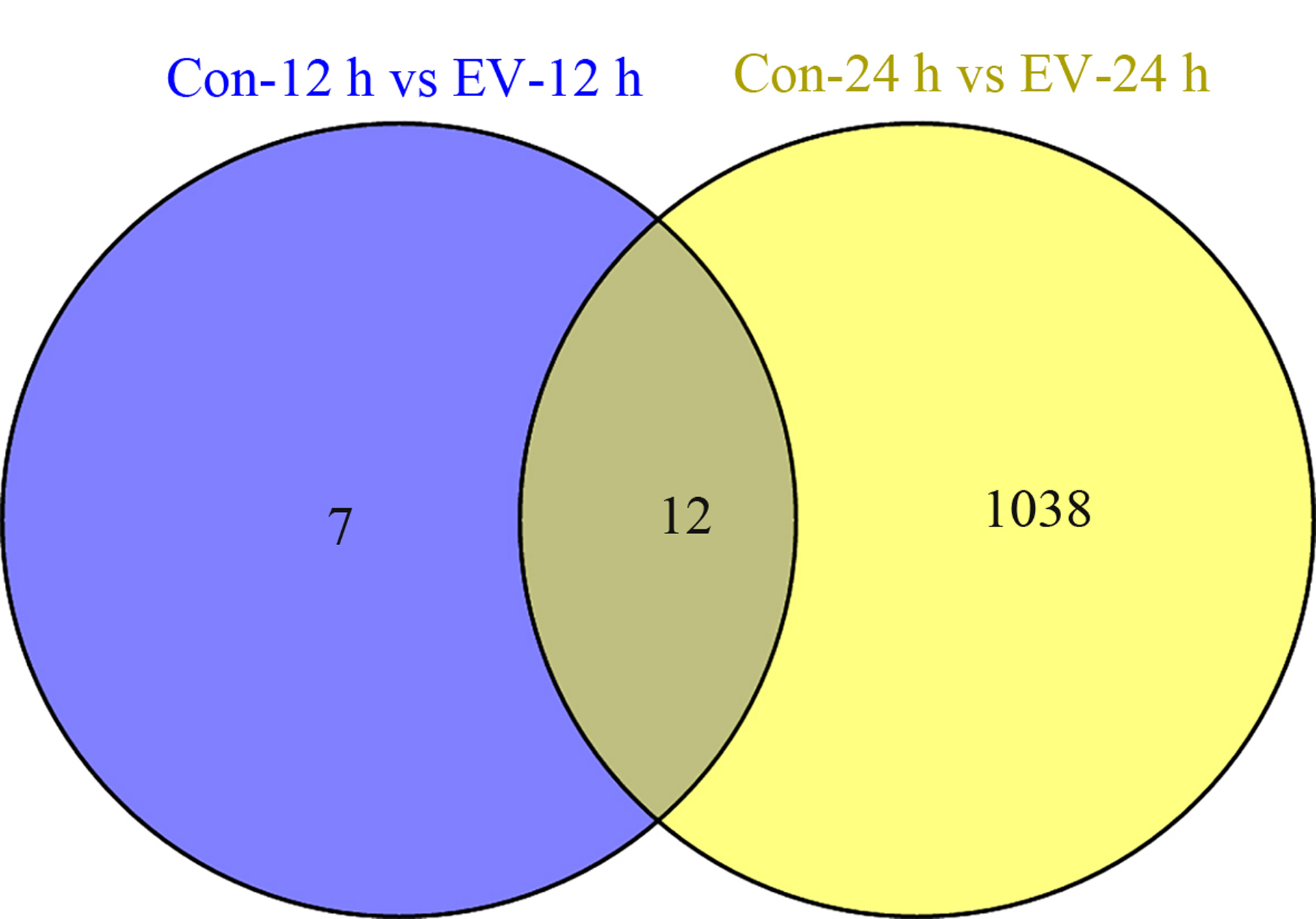

Supplement: Supplementary file 5 — Figure S2. Venn diagram of the up- and down-regulated genes identified following comparisons of the Con-12 h vs. EV-12 h and Con-24 h vs. EV-24 h treatment groups. (JPG 287 kb) [file 12917_2018_1721_MOESM5_ESM.jpg]
